# CSFV Infection Up-Regulates the Unfolded Protein Response to Promote Its Replication

**DOI:** 10.3389/fmicb.2017.02129

**Published:** 2017-11-02

**Authors:** Wencheng He, Hailuan Xu, Hongchao Gou, Jin Yuan, Jiedan Liao, Yuming Chen, Shuangqi Fan, Baoming Xie, Shaofeng Deng, Yangyi Zhang, Jinding Chen, Mingqiu Zhao

**Affiliations:** College of Veterinary Medicine, South China Agricultural University, Guangzhou, China

**Keywords:** CSFV, ER stress response, UPR signaling pathways, IRE1, XBP1, GRP78, replication

## Abstract

Classical swine fever (CSF) is an OIE-listed, highly contagious animal disease caused by classical swine fever virus (CSFV). The endoplasmic reticulum (ER) is an organelle in which the replication of many RNA viruses takes place. During viral infection, a series of events elicited in cells can destroy the ER homeostasis that cause ER stress and induce an unfolded protein response (UPR). In this study, we demonstrate that ER stress was induced during CSFV infection as several UPR-responsive elements such as XBP1(s), GRP78 and CHOP were up-regulated. Specifically, CSFV transiently activated IRE1 pathway at the initial stage of infection but rapidly switched off, likely due to the reduction in cytoplasm Ca^2+^ after viral incubation. Additionally, our data show that the ER stress induced by CSFV can promote CSFV production, which the IRE1 pathway play an important role in it. Evidence of ER stress *in vivo* was also confirmed by the marked elevation of GRP78 in CSFV-infected pig PBMC and tissues. Collectively, these data indicate that the ER stress was induced upon CSFV infection and that the activation of the IRE1 pathway benefits CSFV replication.

## Introduction

The ER is a extensive cellular membranous structure that provides a special environment for lipid biosynthesis, protein folding and secretion, and calcium homeostasis (Healy et al., 2012). A variety of external stimuli, such as chemical insult, glucose deprivation and pathogen invasion have been shown to disrupt the ER homeostasis, leading to its failing to perform the normal functions described above, these cause ER stress and trigger subsequent cellular signaling pathways termed the unfolded protein response (UPR) to restore ER homeostasis (Fang and Shen, 2004; Schröder and Kaufman, 2005; Agostinis and Afshin, 2012).

The UPR is comprised of three branches: IRE1 (inositol-requiring enzyme 1), PERK (PKR-like ER protein kinase), and ATF6 (activating transcription factor 6). Under normal conditions, these three sensor proteins are sequestered by 78 KD glucose-regulated protein (GRP78), an ER chaperone that acts as a master regulator of the UPR and the up-regulation of which reflects the activation of the UPR program. Upon activation, IRE1 leads to phosphorylation and stimulates its endonuclease activity, which not only cleaves nascent mRNAs destined for the ER [pathway named regulated IRE1-dependent decay (RIDD)], but also cleaves 26-nucleotide intron from X-Box binding Protein-1 (XBP1) mRNA. The spliced form of XBP1 [XBP1(s)] encodes a protein acts as a potent transcriptional activator of many genes including chaperones, phospholipid biosynthesis enzymes and the ER degradation-enhancing alpha-mannosidase-like protein (EDEM), which is involved in the ER-associated protein degradation (ERAD) pathway. The activation of the PERK pathway results in the phosphorylation of the eukaryotic translation initiation factor 2 subunit (eIF2α), leading to translation attenuation; however, p-eIF2α can selectively activate the expression of activating transcription factor 4 (ATF4), which up-regulates the genes involved in restoring ER redox homeostasis. For the ATF6 pathway, activated ATF6 migrates to the Golgi apparatus where it is cleaved by S1P and S2P proteases, releasing a soluble fragment that enters the nucleus and activates the transcription of ER chaperones and other genes related to ER homeostasis. However, under serious ER stress, UPR can induce apoptosis for the benefit of the entire organisms (Zhang and Kaufman, 2004; Malhotra and Kaufman, 2007; Bravo et al., 2013).

Classical swine fever (CSF) is an Office International des Epizooties (OIE)-listed, highly contagious disease in swine that is caused by classical swine fever virus (CSFV), which belongs to the *Pestivirus* genus within the *Flaviviridae* family (King, 2012; Mehlhorn, 2015). The disease is distributed nearly worldwide; at present, many Asian and Latin American countries, as well as some countries in Eastern Europe are CSF-endemic regions, CSFV poses a great threat to pig husbandry as the differing virulence of CSFV strains make a highly variable clinical picture of CSF and the immunosuppression and immune evasion features of this virus lead to a persistent infection of this disease (Chander et al., 2014; Ji et al., 2015).

*Flaviviridae* members utilize host cell membranes to construct organelle-like structures to establish the appropriate platform for viral replication. The main source of these membranes is provided by the ER, where polyprotein biogenesis and virion formation take place (Uchil and Satchidanandam, 2003; Gillespie et al., 2010; Paul and Bartenschlager, 2013; Paul and Bartenschlager, 2015; Tautz et al., 2015). This process may damage ER homeostasis and induce ER stress (Blázquez et al., 2013; Chan, 2014a; Jheng et al., 2014). Although the UPR performs the ER homeostasis recover function that is essential for cell survival, the outcome of UPR activation to viral infection maybe more complicated. On the one hand, the ER expansion and increased chaperone expression promoted by UPR may benefit viral replication and viral protein folding; on the other hand, translational attenuation, ERAD, RIDD and apoptosis, which are also mediated by UPR, may limit viral replication (Zhang and Wang, 2012; Ambrose et al., 2013). Thus, it is not surprising that many viruses have evolved different strategies to customize or manipulate the UPR program for their own benefit (He, 2006; Ambrose and Mackenzie, 2011; Lyoo et al., 2015). Thus far, a number of documents have reported that the CSFV life cycle was associated with ER (Tang et al., 2010; Guo et al., 2011; Gladue et al., 2014; Aguilella et al., 2016; Largo et al., 2016). Nevertheless, the significance and associated mechanisms of UPR in CSFV infection remains largely unexplored.

In this study, through quantitative polymerase chain reaction (q-PCR) and immunoblotting analyses, we found that several UPR-response elements, such as XBP1(s), GRP78 and c/EBP homologous protein (CHOP), were up-regulated upon CSFV infection. Specifically, CSFV transient activated IRE1 pathway during or soon after virion entry. We also found that cytoplasm Ca^2+^ was down-regulated after viral incubation, which may explain the IRE1 activation. Additionally, using the ER stress inducer or inhibitor to regulate the cellular ER stress level, we found that the ER stress induced by CSFV infection can promote CSFV replication, which benefit from the activation of the IRE1-XBP1-GRP78 signal. These results add valuable information to understanding the relationship between the CSFV and UPR components and will help elucidate the consequences of these pathways for CSFV pathogenesis.

## Materials and Methods

### Antibodies, Chemicals and Plasmids

The primary antibodies used in the study were specific for GRP78 (Santa Cruz Biotechnology, sc-13968), GRP94 (Novus, NBP2-44690-0.02mg), eIF2α (phospho-S51) (Bioworld, BS4787), eIF2α (Bioworld, BS3651), IRE1 (phosphor S724) (Abcam, ab48187), IRE1 (Bioss, bs-8680R), ATF6 (ABclonal, A0202), ATF4 (ImmunoWay, YT1102), CHOP (Santa Cruz Biotechnology, sc-166682), CSFV-E2 (JBT, 2229), CSFV-Npro (a gift from Hunan agricultural university professor YuXingLong), Tubulin (Beyotime, AT819), and Actin (Beyotime, AT0003). The secondary antibodies Goat anti-Mouse IgG-HRP (Bioworld, BS12478) and Goat anti-Rabbit IgG-HRP (Bioworld, BS13278) were purchased from Bioworld Technology. Thapsigargin (Calbiochem^®^, 67526-95-8), 4μ8c (selleck.cn, S7272) and BIX (Sigma, SML1073) were dissolved in dimethyl sulfoxide (DMSO) at the indicated concentrations. Tauroursodeoxycholic acid (sodium salt, Merck-Millipore, 580549-1GM) and NH_4_CL (Damao Chemical Reagent, 12125-02-9) were dissolved in ddH_2_O at the indicated concentrations. Fluo4-am (BestBio, BB-48113) was diluted 50-folds with Hank’s Balanced Salt Solution (HBSS). pEGFP-N_1_ and pEGFP-GRP78 were prepared in our laboratory. Additionally, shRNA vector targeting the coding sequences of GRP78 and the scrambled shRNA were designed by and purchased from Cyagen.

### Cells, Virus, and Animal

The swine kidney cell line PK-15 was maintained in complete Dulbecco’s modified Eagle’s medium (DMEM) supplemented with 10% (v/v) fetal bovine serum (FBS) and penicillin-streptomycin (100 IU/ml and 100 μg/ml, respectively, Gibco). The cells were grown at 37°C in a humidified incubator with 5% CO_2_. The CSFV strain (Shimen) used in this study was propagated in the PK-15, the virus titer was determined by a 50% tissue culture infectious doses (TCID_50_) assay on PK-15. The multiplicity of infection (MOI) was confirmed according to the virus titer from the PK-15. UV-inactivated CSFV was obtained by irradiating with UV light for 2 h, and infectivity was confirmed by detecting the virus titer as described above. The pseudotyped CSFV particles contains all CSFV structure proteins including C, E_0_, E_1_ and E_2_ was constructed by baculovirus expression vector system and saved in our laboratory previously. PK-15 cells were infected with CSFV at various MOI according to the requirements of different experiments. The mock was infected with phosphate-buffered saline (PBS). The virus was incubated at 37°C for 1 h, the inoculums were removed, and the cells were washed with PBS twice and maintained in DMEM with 2% FBS. The Tibet minipigs used *in vivo* experiments were obtained from Pearl Laboratory Animal Sci. & Tech. Co. Ltd. and housed in our laboratory animal center.

### Sucrose Gradient Separation

The viral culture was collected and concentrated by PEG-6000 precipitation (7.5%) for overnight at 4°C, then collected the PEG-6000 precipitated virus by centrifuging at 10,000 rpm for 30 min at 4°C, re-suspended the pellet in a small volume in PBS. Layered the resuspending on a 20–40% sucrose gradient, the gradients was centrifuged at 35,000 rpm for 4 h at 4°C, after that, removed the 20% sucrose fraction and extracted the band between 20% and 40% sucrose carefully. The extraction was diluted with PBS and centrifuged at 40,000 rpm for 1 h at 4°C to remove the sucrose, the precipitation was collected and re-suspended in a small volume in PBS for later experiments.

### RNA Extraction, q-PCR, and XBP1 Splicing Assays

Real-time q-PCR was used to measure targeted genes expression. Briefly, total RNA was isolated from PK-15 with Trizol reagent (TAKARA, 9108) and reversed to cDNA using PrimeScript^TM^ RT Master Mix (Perfect Real Time, RR036A) according to the manufacturer’s protocol. q-PCR was performed using the SYBR^®^ Premix Ex Taq^TM^ II (TAKARA, RR820A) on an iQ5 iCycler detection system (Bio-Rad). Relative changes in the mRNA levels of genes were analyzed using the 2^-ΔΔC_T_^ method and normalized to the reference gene *GAPDH.* The primers used in study are display in **Table 1**, specifically, the primers for UPR downstream genes detection were spaned the intron to avoid the affect of contaminated DNA during RNA extraction process and the q-PCR results were not effected by any DNA contamination. For virus copies detection, viral RNA was extracted using an E.Z.N.A.^®^ Viral RNA Kit (Omega, R6874-01) and cDNA was synthetized as described above. The cDNA was then amplified using SYBR^®^ Premix Ex Taq^TM^ II (TAKARA, RR820A) with an iQ5i Cycler detection system (Bio-Rad, United States). The primers (see in **Table 1**) targeting a region specific to the CSFV *NS5B* gene were used in this study. The recombinant plasmid pMD18-T containing the *NS5B* gene of CSFV was used to construct a reference curve. To analyze the splicing level of *XBP1*, PCR was performed for 35 cycles [94°C for 30 s, 55°C for 45 s, and 72°C for 60 s (8 min in the final cycle)] using the primers (see in **Table 1**) across the splice site capable of amplifying both the un-spliced [XBP1(u), 474 bp] and spliced form of *XBP1* [XBP1(s), 448 bp], separated on a 3% agarose gels contain ethidium bromide for electrophoresis for 90 min and finally viewed by UV illumination.

**Table 1:** Primers and shRNA used in this study.

Gene	Primer sequence (5′-3′)Application	Application
*ATF6*	F: TTGGGATTCTACCCTGTTTGC	q-PCR
	R: TTTCATAAGTTTCCTTTGCTGC	
*ATF4*	F: TCAGACAACAGCAAGGAGG	q-PCR
	R: ATGGTTTCCAGGTCATCTAT	
*CHOP*	F: CTGG′TGAGGAGGAGTC	q-PCR
	R: CTGGAATCAGGCGAGTGT	
*Calnexin*	F: ACCAAGCCTCTCATTGTTCAG	q-PCR
	R: ATAAGGGGTCTTGTCGTGGAA	
*Calreticulin*	F: ATCTCTGGCAGGTCAAGTCT	q-PCR
	R: TGTCTTTCATTTGCTTTTCTG	
*ERp57*	F: CTGTAAGAACCTGGAGCCCAAGT	q-PCR
	R: TCATTGGCTGTAGCATCCAT	
*EDEM1*	F: TTGACTCTTGTTGATGCATTGGA	q-PCR
	R: GCTTTCTGGAACTCGGATGAAT	
*GAPDH*	R: GCTTTCTGGAACTCGGATGAAT	q-PCR
*GAPDH*	F: TCATGACCACAGTCCATGCC	q-PCR
	R: GGATGACCTTGCCCACAGCC	
*GRP78*	F: CCTACTCGTGCGTTGGGGT	q-PCR
	R: GACGGCGTGATGCGGTT	
*GRP94*	F: GTCCTGCTGACCTTCGGG	q-PCR
	R: GCTTCTTCCTCTCTCTGTACTATTTC	
*XBP1(s)*	F: ′CAGAGTAGCAGCTCAGACTGC	q-PCR
	R: TCCTTCTGGGTAGACCTCTGGGAG	
*XBP1(s)*	F: TCCGCAGCAGGTGCAG	RT-PCR
	R: GGTCCAAGTTGAACAGAATGC	
*XBP1(u)*	F: CGCAGCACTCAGACTACG	RT-PCR
	R: GAAGAGTCAACACCGTCAGA	
CSFV-*NS5B*	F: CCTGAGGACC′CACATGTTG	q-PCR/ RT-PCR
	R: TGGTGGAAGTTGGTTGTGTCTG	
*GRP78*	F: CGGGATCCCGATGAAGCTGTCCCTGGT	Cloning
	R: CCGCTCGAGCGGCTACAACTCATCTTTGTCTGC	
*XBP1*	F: ′CAGAGTAGCAGCTCAGACTGC	RT-PCR
	R: TCCTTCTGGGTAGACCTCTGGGAG	
*shGRP78*	CATTTGCACCAGAAG′TTTCTCGAG′T	RNAi
	TTCTTCTGGTGC′TG	
*Scramble*	CCTAAGGTTAAGTCGCCCTCGCTCGAGCGAG	RNAi
	GGCGACTTAACCTTAGG	

### Calcium Measurements

The intracellular Ca^2+^ levels were measured by the calcium-sensitive dye Fluo4-am using flow cytometry. Briefly, cells were digested and washed 3 times with HBSS, then loaded with Fluo4-am at 37°C for 40 min (protected from light). Next, after adding a 5-fold volume of HBSS with 1% fetal serum further incubated at 37°C for 40 min, the cells were washed with HBSS 3 times. Finally, the intracellular Ca^2+^ level was tested by flow cytometry under a 488 nm wavelength.

### Immunoblotting

The medium with the treated cells was removed and then washed three times with ice-cold PBS. The samples were then lysed for 20 min with RIPA lysis buffer containing proteinase inhibitor on ice. The cell lysates were centrifuged at 14,000 × *g* for 20 min at 4°C, and the supernatant was collected and quantified using a BCA protein assay kit. The samples were boiled for 10 min in 5×SDS-PAGE loading buffer before separated on 10 or 12% SDS-PAGE gels and then transferred onto PVDF membranes (Millipore, 0.45 nm). The membranes were then blocked with 5% powder skim milk or 5% bovine serum albumin in Tris-buffered saline with 0.1% Tween 20 (TBST) and then incubated with primary antibodies against the targeted proteins. After three washes with TBST, each membrane was further incubated with an appropriate secondary antibody conjugated to horseradish peroxidase diluted in TBST at 37°C for 1 h. After three washes with TBST, the bound antibodies were reacted with the ECL Plus kit (Beyotime, P0018). Finally, the protein blots were measured using a luminescent image (Tanon 6600).

### Transfection and Gene Silencing

PK-15 cells grown to 60–70% confluence in 12-well cell culture plates were transfected with *GRP78* shRNA vector and pEGFP-GRP78 eukaryotic expression plasmid using the lip2000 transfection reagent (Invitrogen). Briefly, 1.5–2 μg of vector and 3.75–5 μl of lipofectamine 2000 were diluted in 100 μl Opti-MEM medium in 1.5 ml eppendorf tubes, respectively, stored at room temperature for 5 min, and then the two above-mentioned tubes were gently mixed and further incubated at room temperature for 20 min. The medium was removed and replaced with 1 ml of Opti-MEM containing the transfection mixture and further cultured at 37°C for 4 h, next, the supernatant was replaced with fresh maintenance medium and further incubated for 48 h. The gene knockdown and over-expression efficiency of GRP78 were evaluated by immunoblotting. The scramble shRNA vector and PEGFP-N_1_ plasmid were used as negative controls, respectively.

### Cell Viability Assay

Cell viability was determined by the CCK8 assay. Briefly, cells were seeded in 96-well culture plates and cultured for 24 h at 37°C in a 5% CO_2_ incubator. The medium was replaced with fresh medium containing the corresponding concentrations of Thapsigargin (TG), Tauroursodeoxycholic acid (TUDCA), 4μ8c, BIX or consistent amount of DMSO. After further incubated for the indicated time according to the requirements of the different experiments, the medium was replaced with 100 μL of fresh medium containing 10 μL of CCK8, further incubated for 2 h at 37°C, and then the optical density was measured at 570 nm using the microplate reader (LABEXIM PRODUCTS).

### Statistical Analysis

The data in the study are expressed as the mean ± standard deviation (SD) and were analyzed by student’s *t*-test or one-way ANOVA using the GraphPad prism 5 software. A *P*-value of less than 0.05 was considered statistically significant.

### Ethical Considerations for Animal Experiments

Animal breeding, care and all experiments were performed in adherence to the guidelines of the Laboratory Animal Center of South China Agricultural University and approved by the Animal Ethics Committee.

## Results

### CSFV Infection Activates the Unfolded Protein Response

#### CSFV Transiently Activates the IRE1 Pathway during or Soon after Virion Entry

**FIGURE 1:**
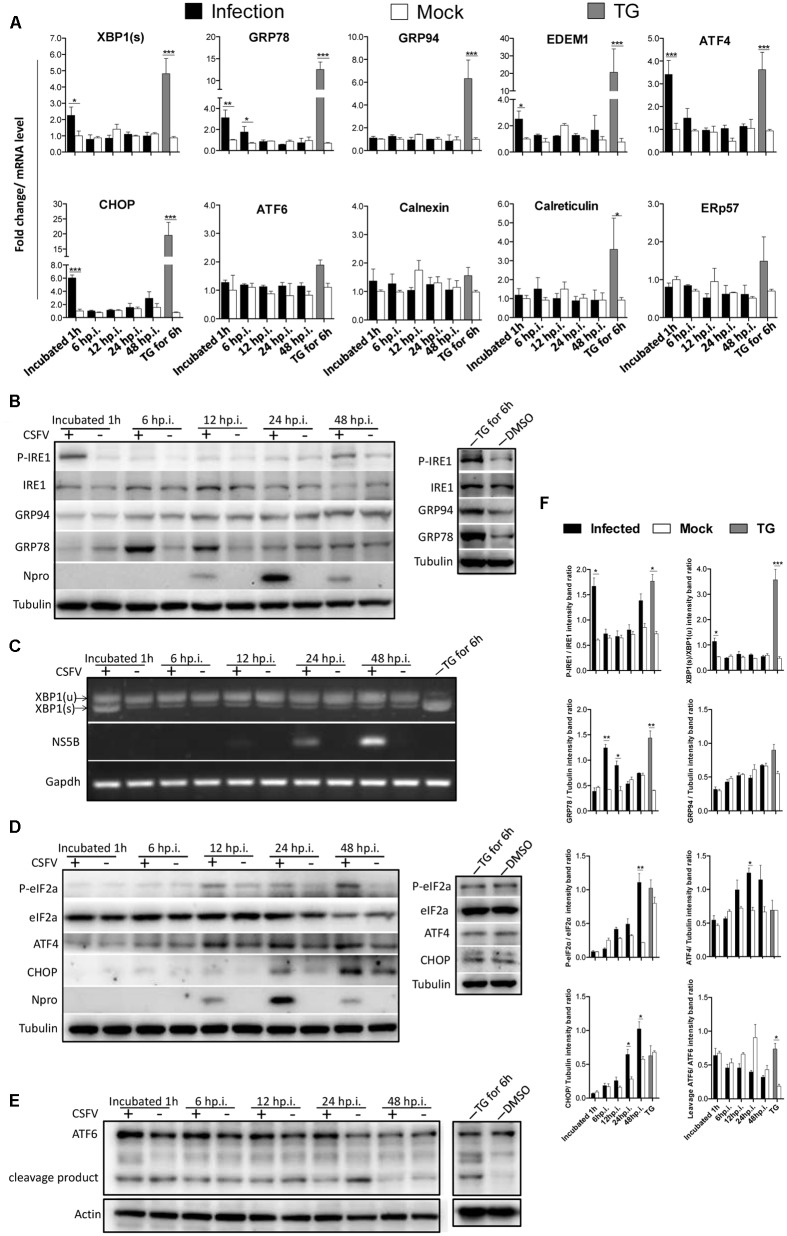
Classical swine fever virus (CSFV) infection induces the activation of UPR. **(A)** RNA extracted from CSFV-infected cells was quantified for the expression of UPR genes Xbp1(s), GRP78, GRP94, EDEM-1, ATF4, ATF6, CHOP, Calreticulin, Calnexin, and ERp57 using q-PCR. Mock-infected PK-15 and Thapsigargin (TG)-treated PK-15 were used as negative and positive controls, respectively, and the fold induction was calculated compared to mock cells at the same time point. Error bars represent the mean ± SD of 3 independent experiments; one-way ANOVA test; ^∗^*P* < 0.05; ^∗∗^*P* < 0.01; ^∗∗∗^*P* < 0.001. **(B,D,E)** Immunoblotting analysis of components of UPR signaling pathways during a time course of CSFV infection. Mock or CSFV-infected PK-15 cells lysates were collected at the indicated time points. Lysates were analyzed for the activation of the IRE1 **(B)**, PERK **(D)** and ATF6 **(E)** pathway by immunoblotting analysis. Tubulin was used as a loading control, and infection was confirmed by detecting the viral protein Npro. Results of a representative experiment of 2 independent experiments are shown. **(C)** RNA was collected as described above, and the splicing levels of XBP1 were analyzed with semi-quantitative PCR as described in materials and methods. The length of Xbp1(u) is 474 bp and Xbp1(s) is 448 bp. **(F)** The relative expression ratios of the targeted proteins/genes were analyzed by densitometric scanning. Error bars represent the mean ± SD of 2 independent experiments; one-way ANOVA test; ^∗^*P* < 0.05; ^∗∗^*P* < 0.01; ^∗∗∗^*P* < 0.001.

To determine whether the UPR is activated upon CSFV infection, PK-15 was infected with CSFV at an MOI of 1. RNA and protein samples of the infected cells were collected at the indicated time points post-infection, and the expression of UPR components was analyzed by q-PCR or immunoblotting. Thapsigargin (TG), a SERCA-pump inhibitor that causes the efflux of calcium from the ER, thus inhibiting calcium-dependent chaperones and increasing protein misfolding, served as a positive control in these assays. The relative expression ratios of the targeted proteins were analyzed by densitometric scanning (**Figure 1F**). As can be observed in **Figure 1A**, the mRNA levels of *XBP1(s)*, *EDEM1* and *GRP78* were transiently elevated more than 2-fold in the CSFV-infected cells after viral incubation; however, this effect was not sustained and was rapidly reversed to basal levels at later time points. According to immunoblotting analysis, we observed that the phosphorylated IRE1 was transiently up-regulated after viral incubation (**Figure 1B**); meanwhile, the spliced *XBP1* was also increased after virus incubation and rapidly switched off, as consistent with IRE1 activation (**Figure 1C**). GRP78, the major indicator of ER stress can be induced by XBP1(s) or ATF6, was up-regulated early at 3 h.p.i. (data not shown), with a peak at 6 h.p.i. and a gradual reduction to basal levels at later stages of infection (24 h.p.i, **Figure 1B**), strongly suggesting that the IRE1-XBP1 signal is responsible for the up-regulation of GRP78. However, when we analyze the expression of GRP94, another ER resident chaperone, we did not find it to be up-regulated compared with mock cells (**Figure 1B**). Notably, the phosphorylated IRE1 was also slightly up-regulated at48 h.p.i., but it did not trigger XBP1 activation.

**FIGURE 2:**
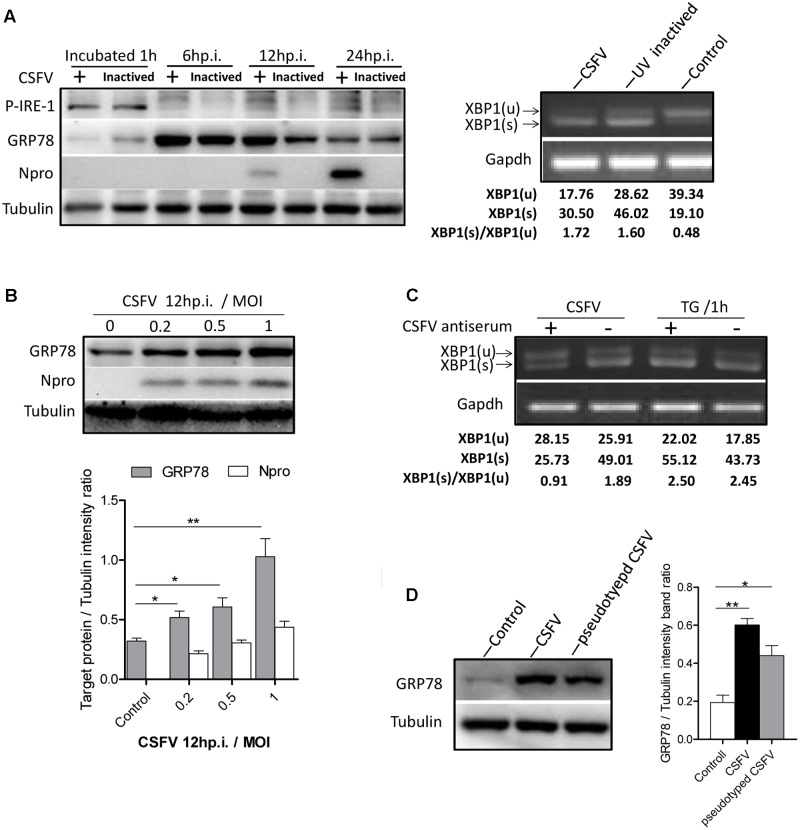
IRE1 pathway is specifically activated by CSFV. **(A)** PK-15 cells incubated with CSFV or UV-inactivated CSFV for 1 h at 37°C; cell lysates and RNA were collected at the indicated time points. Lysates were analyzed for the activation of the IRE1 signal by immunoblotting, and the splicing levels of XBP1 were analyzed by semi-quantitative PCR as described in materials and methods. Tubulin and *GAPDH* were used as loading controls, respectively, and infection was confirmed by the detection of the viral protein Npro. The band intensities of p-IRE1 and GRP78 were determined by densitometry and normalized to the intensities of the corresponding IRE1 or Tubulin bands. The splicing level of XBP1 was calculated as the intensity of XBP1(s) divided by the total intensities of XBP1(u). **(B)** PK-15 cells were infected with CSFV at different MOI (0, 0.2, 0.5, and 1). After 12 h post infection, immunoblotting analysis was performed of the expression of GRP78. Tubulin was used as a loading control, and infection was confirmed by the detection of the viral protein Npro. Error bars represent the mean ± SD of 2 independent experiments; one-way ANOVA test; ^∗^*P* < 0.05; ^∗∗^*P* < 0.01. **(C)** CSFV (MOI = 1) were incubated with a positive (titer > 1:64) or negative specific antiserum against CSFV for 1 h at 37°C, then PK-15 were incubated with the mixture of virus and antiserum. After 1 h of incubation, RNA was collected, and the splicing levels of XBP1 were analyzed by semi-quantitative PCR. Results of a representative experiment of 2 independent experiments are shown. **(D)** PK-15 cells were infected with CSFV or pseudotyped CSFV. After 6 h post infection, immunoblotting analysis was performed of the expression of GRP78. Tubulin was used as a loading control. Error bars represent the mean ± SD of 2 independent experiments; one-way ANOVA test; ^∗^*P* < 0.05; ^∗∗^*P* < 0.01.

As most viruses activate UPR during their biosynthesis, interestingly, we found that the UV-inactivated CSFV could also activate the IRE1-XBP1 signal in a similar manner to the live CSFV (**Figure 2A**). Given that the activation of the IRE1 pathway was observed after CSFV incubation, we attempted to rule out the possibility that the activation of the IRE1 signal was non-specifically generated by the viral supernatant. To test this, first, CSFV were purified through sucrose gradient separation, then PK-15 cells were infected with purified CSFV at different MOI (0, 0.2, 0.5, and 1). After 12 h post infection, immunoblotting showed that the cells infected with CSFV at a high MOI induced a higher level of GRP78 (**Figure 2B**). Additionally, the virus was neutralized by positive anti-CSFV serum that inhibited *XBP1* splicing (**Figure 2C**). Furthermore, the pseudotyped CSFV, which contain all structure proteins of CSFV, could also induce GRP78 up-regulation (**Figure 2D**). These results suggest that the activation of the IRE1 signal was specifically induced by CSFV but not by the viral supernatant.

**FIGURE 3:**
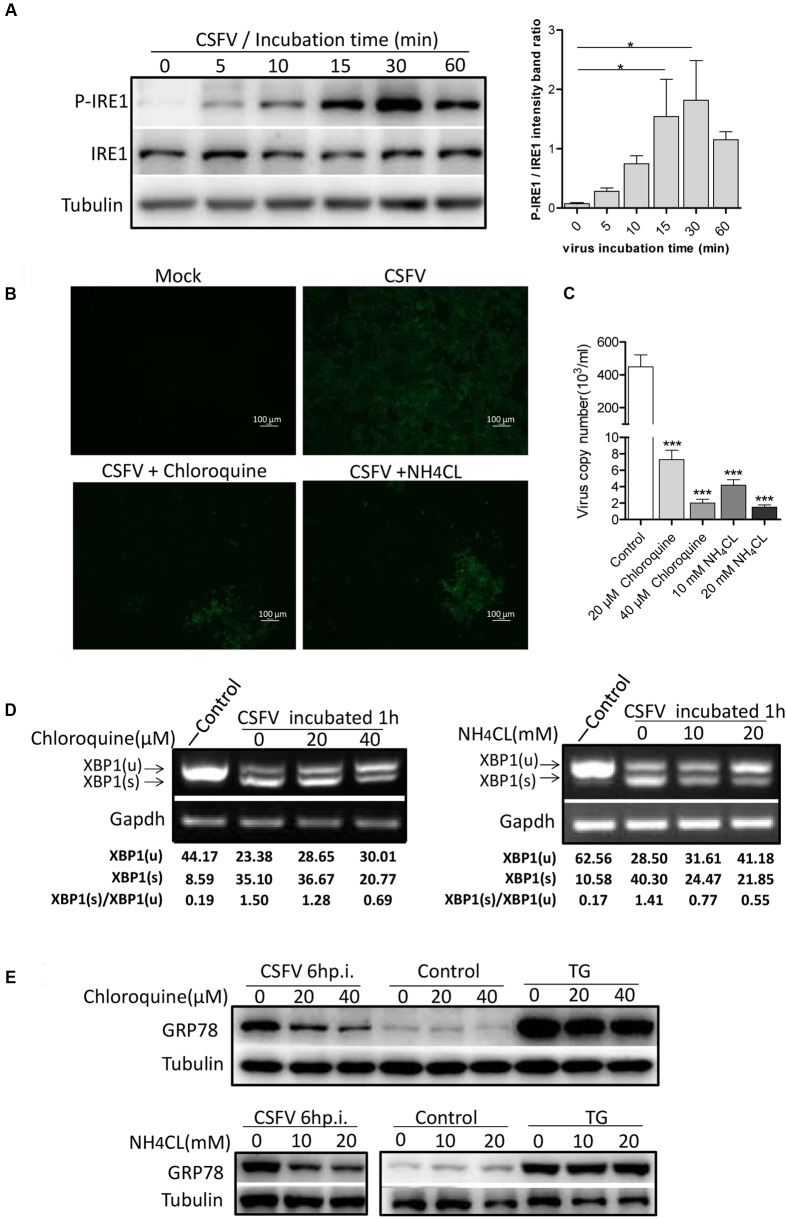
Classical swine fever virus induces the activation of the IRE1 pathway during or soon after virion entry. **(A)** PK-15 cells incubated with CSFV for different time periods (0, 5, 10, 15, 30, and 60 min) at 37°C. Cell lysates were collected at the indicated time points and analyzed for the phosphorylation level of IRE1 by immunoblotting. Error bars represent the mean ± SD of 2 independent experiments; one-way ANOVA test; ^∗^*P* < 0.05. **(B–E)** PK-15 cells were pretreated with NH_4_CL (0, 10, and 20 mM) or Chloroquine (0, 20, and 40 nM) for 3 h, then incubated with CSFV (MOI = 1) combined with NH_4_CL or Chloroquine at corresponding concentrations for 1 h. The inhibitory effect of virus entry by NH_4_CL or Chloroquine were analyzed by the indirect immunofluorescence **(B)** and q-PCR **(C)**. The cell RNA were collected after incubation, and cell lysates were collected after 6 h post infection. The splicing levels of XBP1 were analyzed by semi-quantitative PCR as described in Materials and Methods **(D)** and the expression of GRP78were analyzed by immunoblotting **(E).** Results of a representative experiment of 2 independent experiments are shown.

To determine the exact mechanism of IRE1 activation, we incubated PK-15 cells with CSFV for different durations (0–60 min). The level of phosphorylated IRE1 was observed to increase progressively, starting with 5 min after exposure to CSFV, peaking at 30 min, and then declining at 60 min (**Figure 3A**). Considering the kinetics of IRE1 phosphorylation, we speculated that IRE1 activation was induced during or soon after the process of virion entry. To test this, we treated PK15 cells with well-characterized lysosome tropic agent Chloroquine or NH_4_Cl, which inhibited endosomal acidification, thereby blocking the pH-dependent fusion between the virus envelope and the endosomal membrane, thus inhibiting CSFV entry (Wang et al., 2004; Shi et al., 2016). The indirect immunofluorescence and q-PCR analysis showed that the Chloroquine or NH_4_Cl could inhibit CSFV entry effectively (**Figures 3B,C**), as a result, the splicing level of *XBP1* and the expression of GRP78 were gradually down-regulated as the concentration of Chloroquine or NH_4_CL increased (**Figures 3D,E**), suggesting that the activation of the IRE1 pathway was induced during or soon after virion entry.

**FIGURE 4:**
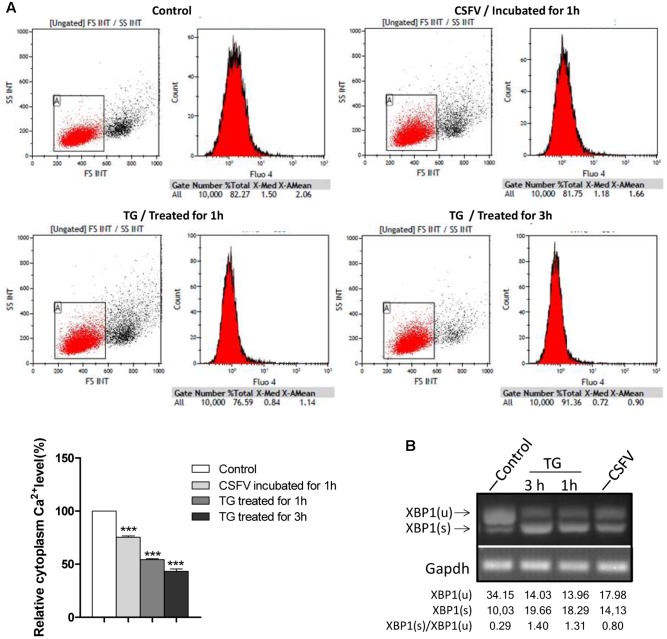
Classical swine fever virus infection regulates cytoplasm Ca^2+^ flux. **(A)** PK-15 were incubated with DMEM or CSFV for 1 h and then digested and washed three times with HBSS, then loaded with Fluo4-am as described in Section “Materials and Methods,” followed by Ca^2+^ flux testing by flow cytometry. Cells were treated with 0.1 μM TG for 1 or 3 h as a positive control. The columnar section in the lower-left corner of the picture is the statistical analysis of the cytoplasm Ca^2+^ level after incubated with CSFV or treated with TG, error bars represent the mean ± SD of 3 independent experiments; one-way ANOVA test; ^∗∗∗^*P* < 0.001. **(B)** The splicing level of *XBP1* in TG-treated cells were analyzed by semi-quantitative PCR.

Aberrant cellular Ca^2+^ regulation is one of important contributors toward ER stress, and the activation of the IRE1 pathway induced by CSFV occurred as quickly as that observed in response to TG; thus, we wanted to determine whether the cytoplasm Ca^2+^ flow changed upon CSFV entry. To test this, we performed cell loading with Flou4-am dye for cytoplasm Ca^2+^ flow detection using flow cytometry analysis. We found that the cytoplasm Ca^2+^ level was down-regulated (by approximately 23%) after CSFV incubation (**Figure 4A**), which was similar to the cell after treatment with TG (0.1 μM, treated for 1 or 3 h), and the trend of reduction in cytoplasm Ca^2+^ demonstrated similar kinetics in the splicing level of *XBP1* in each treatment (**Figure 4B**), suggesting that the reduction in cytoplasm Ca^2+^ after CSFV incubation may contribute to the activation of IRE1.

#### CSFV Activates Downstream Genes as a Product of the PERK Pathway

To determine whether the PERK pathway was activated by CSFV infection, RNA and protein samples were collected at the indicated time point post infection as described above. The q-PCR result showed that the translation level of ATF4 and CHOP were significantly up-regulated after viral incubation (**Figure 1A**). According to immunoblotting analysis, we found that the level of phosphorylated eIF2α was up-regulated at later stage of infection, and the ATF4 and CHOP were both up-regulated at the time coinciding with eIF2α activation (**Figure 1D**), suggesting that the p-eIF2α-ATF4-CHOP signaling was activated by CSFV infection; however, we could not find a suitable antibody against p-PERK in PK-15 cells. Notably, the eIF2α was regulated by three other kinases, including the general control non-derepressible-2 kinase (GCN2), heme-regulated eIF2α kinase (HRI) and Protein Kinase R (PKR), and we previously confirmed that the phosphorylated eIF2α could be activated by the PKR signal upon CSFV infection (Liu et al., 2015).

#### ATF6 Pathway Is Slightly Repressed in CSFV Infection

To determine whether the ATF6 pathway was activated by CSFV infection, RNA and protein samples were collected at the indicated time point post infection as described in 2.1. We compared the mRNA level of endogenous *ATF6* and the chaperones *calnexin*, *calreticulin* and *ERp57* greatly induced by ATF6 in CSFV-infected PK-15 cells with time-matched controls using q-PCR (Okada et al., 2002). We did not find obvious differences in those genes between the infected and mock cells (**Figure 1A**); however, immunoblotting analysis show that the expression of cleaved ATF6 was somewhat decreased at the mid stages of infection compared with the control (**Figure 1E**), suggesting that the ATF6 pathway was slightly repressed in CSFV infection. Further studies should investigate the interaction between ATF6 and CSFV to confirm this notion.

### The ER Stress Is Induced by CSFV Facilitates CSFV Replication

**FIGURE 5:**
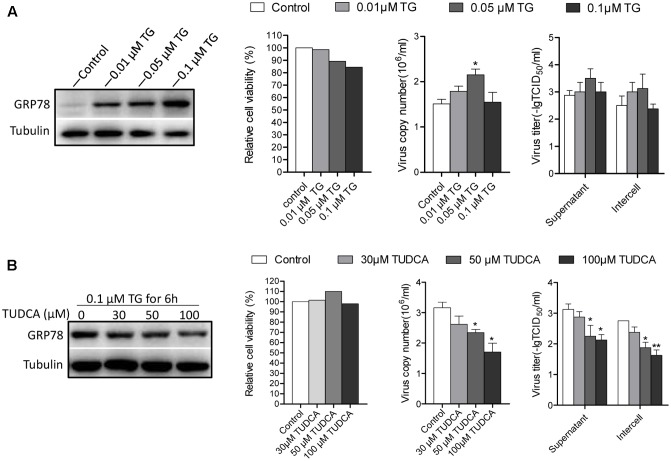
Chemically modified ER stress affects CSFV replicaton. **(A)** PK-15 cells were pretreated with TG (0, 0.01, 0.05, and 0.1 μM) for 6 h. The medium was then removed and infected with CSFV. Twenty four hour post-infection, cell freeze-thaw and cell supernatant were collected for detecting the viral titer and copies using indirect immunofluorescence and q-PCR, respectively. The ER stress level affected by TG was analyzed by detecting the expression of GRP78. **(B)** PK-15 cells were pretreated with TUDCA (0, 30, 50, and 100 μM) for 6 h and then infected with CSFV supplemented with TUDCA at corresponding concentrations during the course of infection. After 24 h post infection, samples were collected, and the viral titers and copies were detected as described above. The ER stress-inhibiting effect by TUDCA was analyzed by detecting the expression of GRP78. Error bars represent the mean ± SD of 2 independent experiments; one-way ANOVA test; ^∗^*P* < 0.05.

Given that the UPR program was induced upon CSFV infection, we were interested in investigating the effects of ER stress on CSFV replication. PK-15 cells were treated with the ER stress inducer TG and inhibitor TUDCA for 6 h at different concentrations to regulate the cell ER stress level before infection with CSFV. The TUDCA were maintained in medium during the course of infection to inhibit ER stress induced by CSFV, while the medium containing TG were removed after viral incubation. The viral replication was assessed for viral RNA and infectious virions at 24 h post infection using q-PCR and indirect immunofluorescence. Our data show that the cells treated with a low concentration of TG (0.01 and 0.05 μM) produced more viral copies and titers compared with the control; however, as the concentration of TG increased to 0.1 μM, under intense ER stress circumstances, the viability of cells reduced (∼18% reduction), and both of the viral copies and titer were slightly reduced (**Figure 5A**). Additionally, the cells treated with TUDCA (at concentrations of 30, 50, and 100 μM, none of the TUDCA treatments had an obvious effect on cell viability) to inhibit ER stress induced by CSFV also showed a reduction in viral production (**Figure 5B**). These data show that the ER stress induced by CSFV infection (an certain extent but not intense ER stress), promotes CSFV yield, while inhibiting this stress response could suppress CSFV production.

### The Activation of IRE1 Pathway Benefits CSFV Replication

**FIGURE 6:**
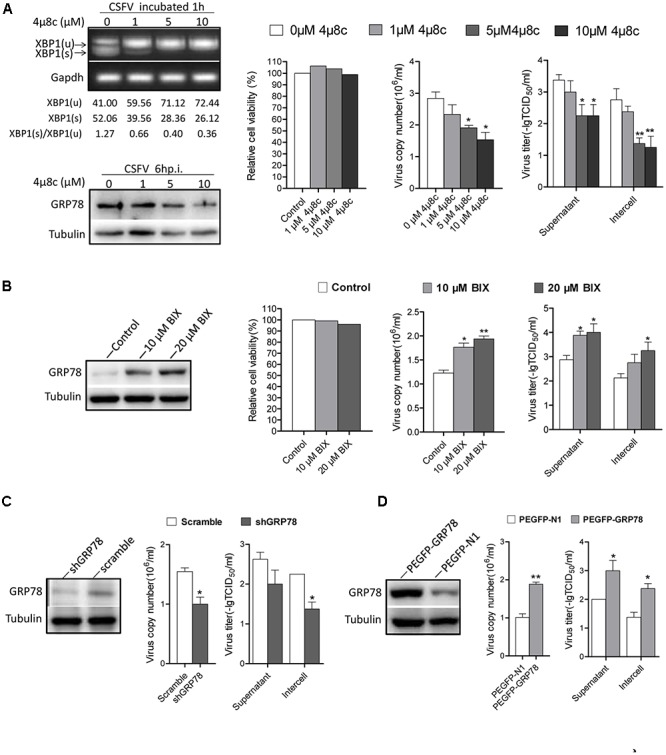
The activation of IRE1 pathway promotes CSFV replication. **(A)** PK-15 cells were pretreated with 4μ8c (0, 1, 5, and 10 μM) for 6 h then infected with CSFV containing the corresponding concentrations of 4μ8c. After 24 h post infection, samples were collected and viral titers and copies were detected as described above. The inhibiting effect of 4μ8C on the IRE1-XBP1 signal was analyzed by detecting the splicing level of XBP1 and the expression of GRP78. Error bars represent the mean ± SD of 2 independent experiments; one-way ANOVA test; ^∗^*P* < 0.05, ^∗∗^*P* < 0.01. **(B)** PK-15 cells were infected with CSFV, after 12 h post infection, BIX (0, 10, or 20 μM) was added to cell medium to up-regulate the expression of GP78, after 24 h post infection, samples were collected and viral titers and copies were detected as described above. Error bars represent the mean ± SD of 2 independent experiments; one-way ANOVA test; ^∗^*P* < 0.05, ^∗∗^*P* < 0.01. **(C)** PK-15 was transfected with the shGRP78 or shRNA-Scramble eukaryotic expression plasmid followed by CSFV infection. Samples were collected for viral titers and copies were detected as described above. Error bars represent the mean ± SD of 2 independent experiments; student’s *t*-test; ^∗^*P* < 0.05. **(D)** PK-15 were transfected with the PEGFP- GRP78 or PEGFP-N1 eukaryotic expression plasmid followed by CSFV infection. After 24 h post-infection, samples were collected for viral titers, and copies were detected as described above. Error bars represent the mean ± SD of 2 independent experiments; student’s *t*-test; ^∗^*P* < 0.05, ^∗∗^*P* < 0.01.

As the IRE1 signal was induced early after CSFV incubation, we were interested in investigating the effects of IRE1 signal on CSFV replication. First, we sought to determine the consequences of CSFV replication if the signal of IRE1 was blocked. Treating cells with 4μ8c, a specific IRE1 endonuclease inhibitor that blocks the IRE1-XBP1 signal (Cross et al., 2012). Therefore, CSFV could not activate the downstream signal of this pathway during infection. Our data show that the virus copies and titers were gradually reduced followed by an increase in the concentration of 4μ8c (**Figure 6A**).

GRP78 is an important element of UPR and it up-regulated obviously upon CSFV infection (3–18 h.p.i), treating cells with BIX, which act as a potent inducer of GRP78 (Kudo et al., 2008), we found that the cells treated with BIX promote CSFV yield compared with control (**Figure 6B**), Additionally, the RNAi technology used to knock down the GRP78 expression also showed a lower viral production compared with the control (**Figure 6C**). While the cells transfected with PEGFP- GRP78 eukaryotic expression plasmid to enhance the expression of GRP78 produced more virus copies and higher titer compared with the control (**Figure 6D**). These results indicated that the activation of the IRE1-XBP1-GRP78 signal, particularly the up-regulation of GRP78, promote CSFV production.

### GRP78 Is Up-Regulated in CSFV-Infected Pig’s PBMC and Organ Tissue

**FIGURE 7:**
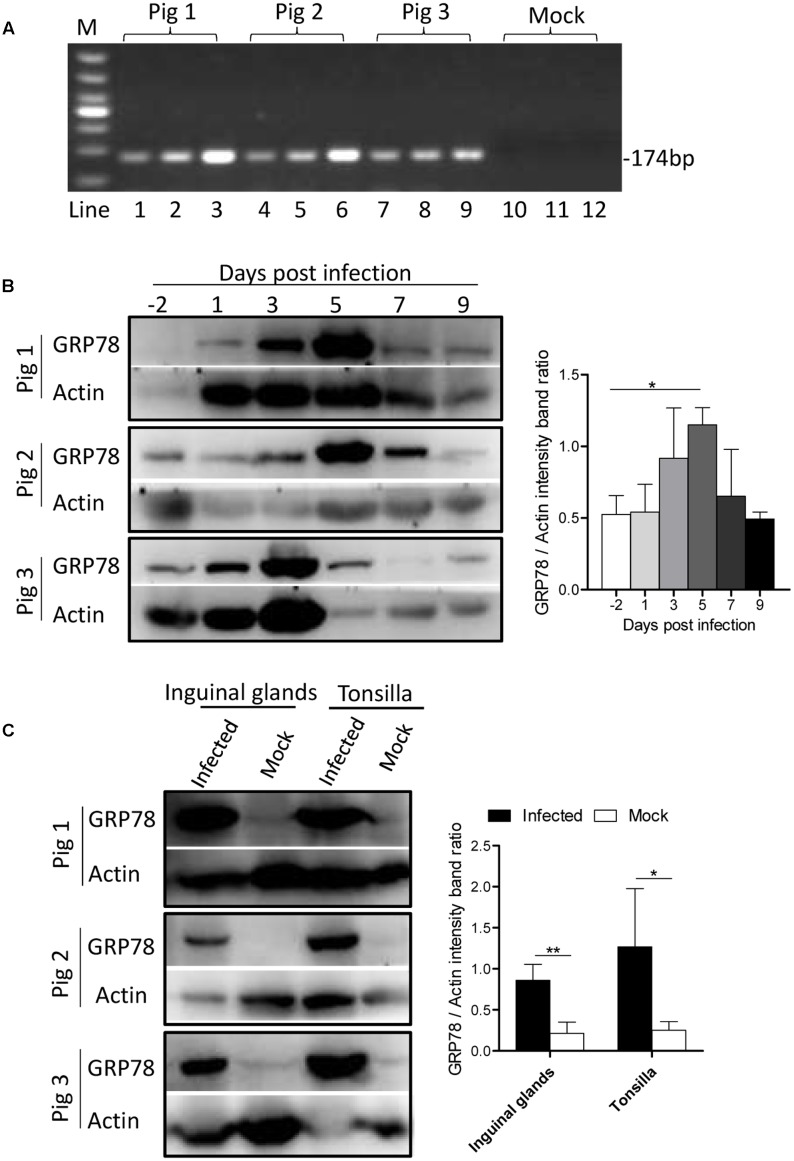
GRP78 is up-regulated in CSFV-infected pig’s tissues and PBMC. **(A)** The PBMC, tonsillar and inguinal gland samples were collected at 9 d.p.i. The viral RNA in those tissues was extracted with a viral RNA kit, and the infection of the experimental animals were detected by RT-PCR using primers specific for viral gene *NS5B*. Lines 1-12 represent the tonsillar (lines 1, 4, 7, and 10), inguinal glands (lines 2, 5, 8, and 11) and PBMC (lines 3, 6, 9, and 12), respectively. **(B)** Three Tibet Minipigs were infected with CSFV (10^5^ TCID_50_) by intramuscular injection. One Tibet Minipig was used as a control. During the course of infection, blood samples were drawn from the experimental animals, and peripheral-blood mononuclear cells (PBMC) was separated at the indicated time points before and post-infection (–2, 1, 3, 5, 7, and 9 d.p.i.). The expression of GRP78 was analyzed by immunoblotting. Error bars represent the mean ± SD of 3 independent experiments; one-way ANOVA test; ^∗^*P* < 0.05. **(C)** The tonsillar and inguinal glands were collected from the infected pigs at the end of the experiment, and tissues were ground in Liquid Nitrogen and lysed with lysis buffer, and the expression of GRP78 was analyzed by immunoblotting. Error bars represent the mean ± SD of 3 independent experiments; student’s *t*-test; ^∗^*P* < 0.05, ^∗∗^*P* < 0.01.

To determine whether ER stress was induced by CSFV infection *in vivo*, three Tibet Minipigs were infected with CSFV by the intramuscular injection with 10^5^ TCID_50_. One Tibet minipig was used as a control. The infectivity of experimental animals were detected by q-PCR (**Figure 7A**). During the course of infection, blood samples were drawn from the experimental animals, and the peripheral-blood mononuclear cells (PBMC) was separated at indicated time points before and post-infection. For animal welfare considerations, the experimental animals were euthanized, and organ tissue was collected at 9th day post-infection. In the immunoblotting analysis, we found that the expression of GRP78 in PBMC was up-regulated early at the 3rd day post-infection with a peak at the 5th day and then a reduction to the normal level following that (**Figure 7B**), this demonstrated similar kinetics *in vitro*. The tonsillar and inguinal glands collected from the infected pigs at the end of the experiment also showed a significantly higher GRP78 expression compared with the control (**Figure 7C**), suggesting that CSFV induced ER stress *in vivo*.

## Discussion

**FIGURE 8:**
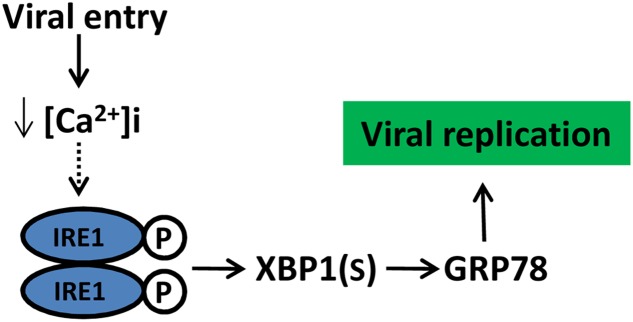
Schematic model of CSFV manipulate UPR and its potential outcome. CSFV activated the IRE1pathway at the initial stage of infection likely due to a reduction in cytoplasm Ca^2+^, the activation of IRE1 pathway benefit viral replication through GRP78 up-regulation. Dotted lines with arrows indicate tentative relationships that warrant confirmation.

In recent decades, ER stress and UPR induced in viral infection have been demonstrated in mammalian cells (Chan, 2014a). The activation of one or more of the three branches of the UPR by the *Flaviviridae* family have been widely reported (He, 2006; Blázquez et al., 2013). In the *Hepacivirus* genus, hepatitis C virus (HCV) infection can activate the three UPR signaling pathways; however, there are contradictory findings regarding the HCV-related suppression of the UPR component (Tardif et al., 2002, 2004, 2005; Chan and Egan, 2005; Chan, 2014b). In the case of the *Flavivirus* genus, Dengue virus (DENV) infection showed a time-dependent activation of the UPR pathways, with PERK activation during the early stages of replication, while the IRE1 and ATF6 activation occurred in the mid and late stages in the replication cycle, respectively (Pena and Harris, 2011). In the *Pestivirus* genus, the bovine viral diarrhea virus (BVDV) activates PERK and eIF2α phosphorylation during viral protein biosynthesis (Jordan et al., 2002). Other members of the *Flaviviridae* family, such as West Nile virus (WNV), Japanese encephalitis virus (JEV), and Tick-borne encephalitis virus (TBEV) activating the UPR component have also been described (Blázquez et al., 2013). These studies highlight that diverse activation of UPR signaling can differ quite significantly between various flaviviruses, it could be inferred that the outcome of UPR signaling may determine the pathogenicity between the different strains of flaviviruses, and indicating a critical role of the UPR regulation in viral infection. Therefore, understanding how and why CSFV regulates UPR will help us further understand the biology and pathogenesis of CSFV (**Figure 8**).

Generally speaking, viruses induce ER stress during viral biosynthesis process as a large amount of immature viral proteins accumulate in host cell that increase the ER burden. Nevertheless, a rare occurrence, such as murine cytomegalovirus, can activate UPR components during virion entry and are enhanced by viral immediate-early or early gene expression (Qian et al., 2012). This, also appears to occur in CSFV infection, as the IRE1 phosphorylation and XBP1(s) were up-regulated soon after exposure to CSFV (whether UV-inactivated or live CSFV), and the pseudotyped CSFV, which contains the structure proteins C, E_0_, E_1_ and E_2_, could also induce GRP78 up-regulation, suggesting that the IRE1 signal was specifically activated by virion itself rather than viral replication. Since the kinetic of the IRE1 phosphorylation are consistent with the process of CSFV entry at that space and time, we treated cells with endosome aicdification inhibitor Chloroquine and NH_4_CLto inhibit the PH-dependent CSFV entry process, as a result, the activation of IRE1 signal was repressed, confirming that the IRE1 signal is induced during or soon after viral entry. Additionally, we found that the cytoplasm Ca^2+^ was reduced by approximately 23% after viral incubation, which was similar to the cell after treatment with TG, suggesting that the decrease in cytoplasm Ca^2+^ may contribute to IRE1 activation. However, on the contrary, cytoplasm Ca^2+^ was up-regulated (approximately 20%) at 24 h post infection (data not shown), this comes as no surprise because CSFV P7 act as a viroporin that modify membrane permeability, in turn leading to Ca^2+^ influx (Aguilella et al., 2016; Largo et al., 2016). This make us question which structural protein of CSFV is involved in cytoplasm Ca^2+^ reductions during or soon after virion entry may contribute to IRE1 activation. We firstly considered the viral core protein, which is supported by evidence from HCV, another member of the *Flaviviridae* family. Its core protein has been shown to trigger ER stress through a mechanism involving ER Ca^2+^ depletion (Benalifuret et al., 2005). we also considered the possibility that the core protein of CSFV may interacts with GRP78 after being release from uncoating, thereby setting IRE1 free from GRP78 and then leading to IRE1 activation; however, we found no protein–protein interactions between the CSFV core protein and the GRP78 through a yeast two-hybrid system (data not shown). Additionally, the expression of GRP78 did not change after cells were transfected with CSFV-core protein eukaryotic expression plasmid (data not shown), suggesting the CSFV core protein is not responsible for the IRE1 activation. Further study should focus on the underlying molecular mechanism by which CSFV regulates the cytoplasm Ca^2+^ level during virion entry and its relationship with IRE1 activation.

Viral infection trigger cellular stress responses, potentially resulting in circumstances that could attenuate viral replication. For example, under ER stress condition, activated PERK phosphorylates eIF2α on Ser51, which may leads to both of the shutoff of cellular and viral mRNA translation to alleviate the ER burden, while the ERAD and RIDD are activated through the IRE1 signal may be a potential threat to viral protein and mRNA degradation (Byun et al., 2014). If the ER stress is too severe to be overcome, the UPR induces cell death through IRE1-mediated activation of JNK and PERK-mediated expression of CHOP, to commit damaged cells to apoptosis for the benefit of the entire organism (Szegezdi et al., 2006; Madeo and Kroemer, 2009). All of those circumstances may suppress viral replication. So, it is not surprising that some viruses have evolved to manipulate UPR to limit harmful aspects of the UPR and promote its persistence in infected cells (Ambrose et al., 2013). For example, the herpes simplex virus (HSV) genome encodes a GADD34 homolog protein which leads to the dephospholyation of eIF2αand overcomes the PERK response (Cheng et al., 2005). The DENV utilizes the IRE1-XBP1 pathway involved in the ERAD program to protect cells from virus-induced cytopathic effects (Umareddy et al., 2007). In general, viral favors the activation of ATF6 and IRE1 pathways as the prosurvival downstream effectors of those signaling; however, in our case, we found that the ATF6 signaling is slightly repressed in at the mid stages of infection, we think that maybe the up-regulation of GRP78 mediated by IRE1 pathway at the early stages of infection that inhibit ATF6 migrates to the Golgi apparatus. In the PERK signaling, our data show that the eIF2α were activated during the mid and late stages of infection; however, *Pestivirus* translation is less sensitive to eIF2α phosphorylation than standard cap-dependent translation because its translation initiation is mediated by the HP (hepatitis C virus/pestivirus) IRES (internal ribosomal entry site), which does not require the ternary complex (Pestova et al., 2008). Besides, our previous studies demonstrated that the activation of the PKR pathway can promote CSFV replication (Liu et al., 2015), suggesting that the activation of eIF2α dose not attenuate to CSFV mRNA translation. Additionally, immunoblotting was performed to analyze the possibility that the activation of IRE1 may contribute to cell apoptosis; however, maybe the transient activation of IRE1 in PK-15 is not adequate enough to stimulate JNK signal, we found no up-regulation in phosphorylated JNK during the course of infection (data not shown), suggesting that the IRE1-JNK-mediated apoptosis pathway was not activated by CSFV, which suppose to favor viral persistent infection. Furthermore, the RIDD-mediated viral mRNA decay process seem to not occur upon CSFV infection because cells treated with 4μ8c inhibiting the endonuclease activity of IRE1 shown a lower viral copies compare with control. As we found the transcription of EDEM 1 (a vital component of the ERAD program) was up-regulated during CSFV infection; however, the potential threat of ERAD has not been determined in this study.

In addition to limiting the harmful aspects of the UPR, some viruses have evolved to take advantage of the UPR for viral production and pathogenesis. For example, HCV-induced chronic ER stress caused by the adaptation of infected hepatocytes to UPR has been considered important for its persistent infection *in vivo* (Merquiol et al., 2011). In our case, it appears to be a strategy for viral infection because the IER1 partway was activated before viral biosynthesis and because blocking a different site of this pathway diminished virus yield. Therefore, understanding the consequence of different levels of ER stress to CSFV production is important. Using pharmacological treatment to regulate cellular ER stress level, we found that certain extent of ER stress could promote the CSFV production even though the viability was somewhat reduced. However, under intense ER stress circumstances, the viral proliferation was slightly depressed. This may explain why the IRE1 pathway was only activated at the beginning of infection, to avoid the harmful sustained activation of UPR, which could potentially attenuate viability and viral replication. Furthermore, cells treated with TUDCA, which inhibits the ER stress induced by CSFV, had a negative effect on viral yield. Taking these results together, we conclude that the ER stress induced by CSFV, particularly the activation of the IRE1 UPR branch, benefits CSFV replication.

GRP78 is a known ER chaperone with the important function of maintaining ER homeostasis. We demonstrated that the GRP78 was up-regulated through IRE1-XBP1 signal in CSFV- infected PK-15 cells, and it also up-regulated significantly *in vivo* after challenge with CSFV, which means that GRP78 plays a role in CSFV infection. Substantial evidences has shown that GRP78 plays a important role in viral infection in many ways. GRP78 may not only act as a chaperone helping viral assembly and egress but also act as a cellular receptor on the surface to enhance viral uptake (Buchkovich et al., 2009; Wati et al., 2009; Wu et al., 2011). As GRP78 itself is a potent anti-apoptotic protein, some viruses exploit it to prevent cell apoptosis (Lyoo et al., 2015). In our case, we showed that cells over-expression or knockdown GRP78 have effect on viral replication, since GRP78 localize in multiple intracellular organelles and regulate a multitude of biological processes to maintain cellular homeostasis (Luo et al., 2006; Ni et al., 2011), we speculate that the cells with elevated GRP78 at the early stage of CSFV infection (3–18 h.p.i.) provide a favorable intracellular environment for viral replication. Besides, cross talk between the UPR and immunity has been recently described, it had been reported that GRP78 interacts with the viral proteins to degrade the heavy chain of major histocompatibility complex (MHC) class I through the ER-associated degradation process, which may participate in cytomegalovirus immune evasion (Hegde et al., 2006). As the expression of GRP78 was significantly up-regulated in lymph tissues and BPMC of CSFV-infected pigs, GRP78 may also participate in immunosuppression or immune evasion in CSFV infection which have been well known as the important features of CSF. Further studies should investigate the particular function of the GRP78 interaction with CSFV.

## Author Contributions

WH, JC, and MZ designed experiments; WH, SD, and YZ carried out experiments; WH, JC, HG, and JY analyzed experimental results and data. JL, SF, BX, and HX assisted with animal experiment. WH and YC wrote the manuscript.

## Conflict of Interest Statement

The authors declare that the research was conducted in the absence of any commercial or financial relationships that could be construed as a potential conflict of interest.
